# Suicide Prevention Using Google Ads: Randomized Controlled Trial Measuring Engagement

**DOI:** 10.2196/42316

**Published:** 2023-04-20

**Authors:** Sandersan Onie, Patrick Berlinquette, Sarah Holland, Nicola Livingstone, Coco Finemore, Nyree Gale, Emma Elder, George Laggis, Cassandra Heffernan, Susanne Oliver Armstrong, Adam Theobald, Natasha Josifovski, Michelle Torok, Fiona Shand, Mark Larsen

**Affiliations:** 1 Black Dog Institute Randwick Australia; 2 BERLIN SEM New York, NY United States

**Keywords:** suicide prevention, suicide, suicidal, self harm, digital advertising, Google Ads, search, suicide hotline, advertise, advertising, campaign, mental health, prevention, digital intervention, online intervention

## Abstract

**Background:**

Studies have shown that individuals may search for suicide-related terms on the internet prior to an attempt.

**Objective:**

Thus, across 2 studies, we investigated engagement with an advertisement campaign designed to reach individuals contemplating suicide.

**Methods:**

First, we designed the campaign to focus on crisis, running a campaign for 16 days in which crisis-related keywords would trigger an ad and landing page to help individuals find the national suicide hotline number. Second, we expanded the campaign to also help individuals contemplating suicide, running the campaign for 19 days with a wider range of keywords through a co-designed website with a wider range of offerings (eg, lived experience stories).

**Results:**

In the first study, the ad was shown 16,505 times and was clicked 664 times (4.02% click rate). There were 101 calls to the hotline. In the second study, the ad was shown 120,881 times and clicked 6227 times (5.15% click rate); of these 6227 clicks, there were 1419 (22.79%) engagements with the site, a substantially higher rate than the industry average of 3%. The number of clicks on the ad was high despite a suicide hotline banner likely being present.

**Conclusions:**

Search advertisements are a quick, far-reaching, and cost-efficient way of reaching those contemplating suicide and are needed despite suicide hotline banners being present.

**Trial Registration:**

Australian New Zealand Clinical Trials Registry (ANZCTR) ACTRN12623000084684; https://www.anzctr.org.au/Trial/Registration/TrialReview.aspx?id=385209

## Introduction

### Overview

#### What Is Already Known on This Topic

Individuals may search for suicide-related terms on the internet prior to an attempt. These search terms may reflect their current cognitive state pertaining to suicide. However, there is currently no intervention targeting search pages.

#### What This Study Adds

This study provides evidence for the high level of reach and engagement of a Google AdWords campaign despite a hotline banner likely being present on the search page. Further, this study provides evidence that this intervention is effective in promoting help seeking, with study 1 showing a high number of people calling the national hotline from the landing page. This study also provides information on the feasibility, reach, speed, and cost of this type of intervention.

#### How This Study Might Affect Research, Practice, or Policy

Although a hotline banner appears when suicide terms are searched, the results suggest that this is not enough as many individuals still engage with the advertisement below the hotline banner. Internet ads may be a rapid, far-reaching, and cost-effective way to reach out to individuals for suicide and across a range of health issues.

### Background

Previous studies have shown that individuals may search for suicide-related terms on the internet prior to an attempt. A recent study showed that in the 60 days prior to a suicide attempt, individuals had searched for terms expressing suicidal ideation and suicide means but also relevant keywords not directly associated with crisis (eg, feeling empty, divorce, and alcohol use) [1]. In addition, studies have found that the increases in the volume of internet searches for suicide-related terms predicted subsequent increases in national suicide rates [2-4]. Thus, targeting internet search engines to intervene in such searches, and connect individuals to help, may be an important avenue for intervention.

Internet search ads can be used to reach out to individuals contemplating suicide, or in crisis, by identifying what keywords people are likely to search, presenting an advertisement on the search page when the keywords are searched, and linking to a landing page with appropriate resources and help-seeking information. The reach and effectiveness of these campaigns can be assessed by measuring the impressions (how many times the advertisement is shown), clicks or click rate (clicks on the advertisement), and conversions (specific behaviors performed on the website) [5]. Note that the party placing the advertisements must determine what behaviors they would like visitors to do on the website, counting these behaviors as conversions.

One reason to use advertisements over organic (or nonadvertisement) searches is that when using organic search, it is not guaranteed that a link will appear first on the search result page. A study found that on average, a link on the search page is twice as likely to be clicked on versus the link directly below it [6], suggesting that individuals are most likely to click and engage with the search result presented first. Further, organic search relies on similarities of the keyword and the page itself; thus, a person who is searching for terms pertaining to “loneliness” may not be shown a link to a page with suicide help. Finally, internet searches are a part of daily life, with 93% of browsing sessions starting on the search page [7]; thus, individuals may be more likely to engage with help if the process by which they access help is a part of their day-to-day behavior.

In some search engines, a hotline number will appear in a prominently displayed banner at the top of the search results if certain suicide-related terms are searched. Reports from the United States have shown that in certain instances, the banners have increased calls to the hotline by 10% [8]. However, there is no public information on what keywords will trigger the banner and whether past search history increases or decreases the likelihood of this banner appearing. Further, a Google spokesperson has said and past research has shown that not all relevant keywords will trigger this hotline banner [8,9]. Research has also shown that not everyone in a suicidal crisis would like to call a hotline, suggesting a need for more diverse offerings [10]. Further, a phenomenon called “banner blindness” is often observed, in which eye-tracking data suggest that individuals will tend to ignore elements on a page in the form of a banner [11].

### Objectives

The overall objective of this research was to investigate patterns of engagement with advertisements and landing pages—that is, the first page shown after a link is clicked—designed for those in crisis and contemplating suicide, which was examined in 2 studies. The first study aimed to assess whether the presence of a Google Ads campaign promoting a crisis line would encourage connection with the crisis service. The aim of the second study was to conduct a 2-arm trial investigating the additional benefits of age tailoring while extending the campaign to include individuals at the precrisis or contemplation phase and assess the different engagement levels of different types of search keywords. Both studies are reported in CONSORT (Consolidated Standards of Reporting Trials)–compliant formats.

## Study 1

### Methods

#### Internet Advertisement Platform

For this study, we used Google Ads, Google’s proprietary search advertisement platform, to promote a crisis line to individuals searching for specific suicide-related terms. Google Ads was chosen as the search engine with the largest market share [12] and thus offers maximum reach. Ads are charged only if the advertisement is clicked, not just for displaying the advertisement. Cost per conversion is the average amount of money spent for a single conversion; there is no further charge per conversion.

Google Ads has several ethical protections in place. The data are only available in aggregate format, and it is not possible to identify individual people who have seen the ad, clicked on the ad, or browsed the landing page website. Although Google Ads may use past browsing history to present advertisements that the individual is likely to engage with, the user can erase all data Google has collected [13] or opt out of personalized advertising [14]. Furthermore, personal information, such as email, is never collected or shared without express permission [15].

#### Ethical Considerations

Ethics approval was not required for this study as only deidentified, pre-existing data are reported in aggregate.

#### Participants

Individuals residing in the United States were eligible to participate.

#### Intervention

**Figure 1:**
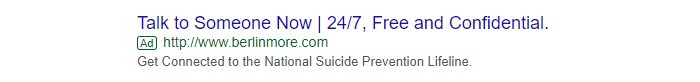
The advertisement that is triggered during the Google Ads campaign in study 1.

An ad campaign was configured to run across the United States for a total of 16 days in 2019 (from October 1 to 3 and from November 15 to 27). The keywords used in this study consisted of 28 commonly searched suicide-related terms that were compiled by a Google Ads agent, an individual who coordinates and runs Google Ads campaigns for organizations. Of the 28 terms, 13 keywords indicated overt suicidality; 7 keywords featured a method or location; 4 pertained to help seeking; and 4 were related to, but not explicitly mentioning, suicide. Note that Google Ads will trigger the ad when a keyword or combination of keywords are semantically similar to the keywords; thus, a single keyword such as “kill myself” may capture a wide range of terms (eg, “end my life” and “take my life”), and a short list may cover a much wider range of keywords. Figure 1 shows the advertisement triggered during the campaign.

If an individual clicked on the link in the ad, it would lead them to a simple landing page consisting of a link to the National Suicide Prevention Hotline for the United States. The “conversion” for this campaign (ie, the desired behavior after an individual had clicked through from the ad to the landing page) was calling the hotline number.

#### Outcomes

Data on impressions, clicks, click rate (clicks/impressions), conversions, conversion rate (conversion/click), cost per click, and cost per conversion were extracted from Google Ads. Total conversion rate was manually calculated (conversions/impressions). The primary outcomes for this trial to measure engagement were click rate (engagement with the ad), conversion rate (engagement with the landing page), and total conversion rate (total engagement with campaign).

#### Statistical Analysis

For any analyses in which we compare rates (eg, click-through rates or conversion rates), we used the MedCalc software (MedCalc Software Ltd), which uses a chi-square test to test a significant incidence rate difference (IRD) [16], set to a significance rate of .05. All rate comparisons in this paper use this method.

### Results

Within the 16-day duration of the ad campaign, the advertisement was shown 16,505 times and was clicked 664 times, yielding a 4.02% click rate. From those who clicked the ad (n=664), there were 101 calls to the hotline, yielding a 15.21% conversion rate with an average cost of US $13.57 per helpline call. The campaign had an overall conversion rate (conversion/impressions) of 0.61% (101/16,505).

To investigate how our campaign performed against industry standards, we conducted a rate comparison test to compare our campaign data versus publicly available industry data. The industry data were drawn from approximately 985,804,416 impressions, 31,250,000 clicks, and 1,171,875 conversions, with a 3.17% click rate and 3.75% conversion rate, collected from the Google Ads agency [17]. The analysis suggested that our campaign overall performed better than the industry standard (IRD 0.004931, 95% CI 0.004405-0.0005457; *P*<.001; ie, 0.49 percentage points higher than the industry standard;).

### Discussion

In study 1, we investigated the efficacy of a Google Ads intervention in encouraging help seeking by calling a helpline by running a campaign across the United States. The results revealed high click-through rates and conversion rates when compared with the industry average across all sectors (click rate: 4.02% vs 3.17% industry average; conversion rate: 15.21% vs 3.75% industry average; overall conversion rate: 0.61% vs. 0.12% industry average), having a 5.08 times greater total conversion rate than the industry average.

As information about when the banner was or was not triggered is limited, it is not possible to provide a further breakdown of the ad engagement based on whether the banner was triggered. Nevertheless, as good engagement was observed with an ad that promoted the national hotline, 2 general conclusions can be drawn. First, if the hotline banner *was* triggered, this suggests that some individuals skipped over the banner but did click on the ad to call the hotline. Second, if the banner was *not* triggered, then the ad led to an individual seeking help that otherwise would not have. Overall, the data provide evidence for the utility of an intervention using Google Ads.

## Study 2

### Background and Objectives

In study 2, we developed a Google Ads campaign designed to be helpful for individuals who may be contemplating suicide but are not in immediate crisis [18]. In this context, we use the term contemplation phase to capture any stages that may lead to a suicide attempt but prior to immediate crisis. Simply providing a link to the hotline may not be the most appropriate response in the contemplation stage, and therefore, we undertook a co-design process to expand what was offered on the landing page. Further, given that the keywords searched may represent different cognitive states, we investigated whether different categories of keywords led to different levels of engagement. Finally, we used Google Ads’ targeting features and investigated whether individuals show higher engagement with landing pages tailored to specific age groups compared to a general all-ages landing page.

### Methods

#### Trial Design

The study used a 2-arm experimental design (landing page: general vs age-tailored—18-24, 25-44, or 45+ years) with 4 initial pathways (individuals searching for different types of keywords: low risk, high risk, help seeking, and means specific). Participants were allocated equally to the 2 arms.

#### Participants

Participants aged >18 years, whose ages could be inferred by Google from their past browsing history and are currently residing in Australia, were eligible to join the trial.

#### Intervention

**Figure 2:**
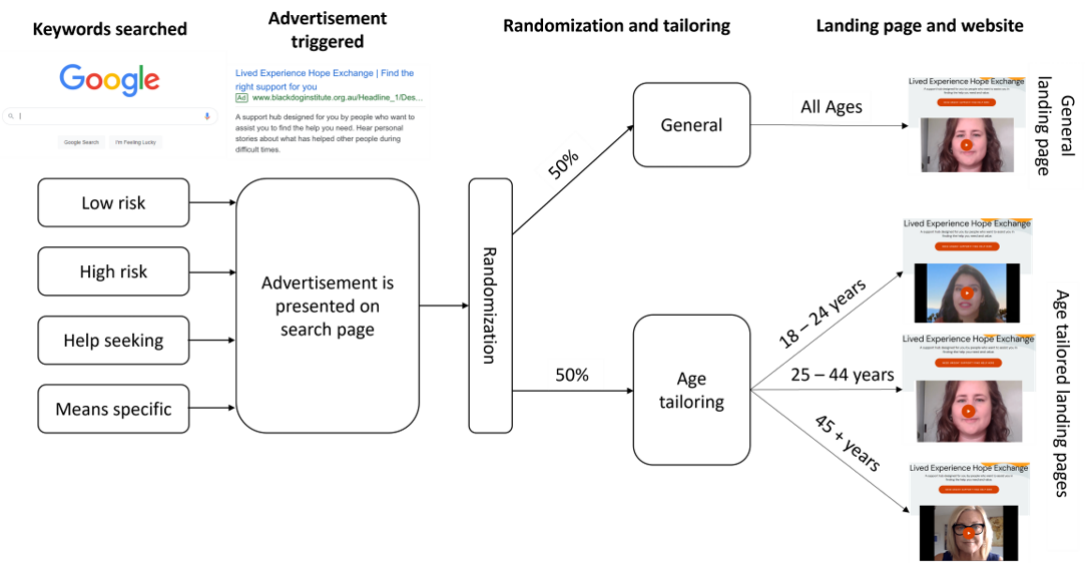
A schematic of the Google AdWords campaign used in this study. As individuals would search for a keyword listed in 1 of 4 keyword lists (low risk, high risk, help seeking or means specific), the advertisement would trigger on the Google search page. If clicked, individuals would be randomly allocated into 1 of 2 study conditions, that is, general or age-tailored landing pages. If in the general condition, individuals would then be presented with the same landing page, whereas if in the age-tailored condition, individuals would be presented with a landing page tailored for their age group.

As with study 1, data were downloaded in an aggregate, deidentified form. The campaign was launched on March 2, 2022, and ran until the prespecified budget was exhausted (March 21, 2022). Full details of the keyword generation, advertisement and landing page co-design process, and content of the landing pages and linked pages can be found elsewhere [10]. A schematic of the campaign can be found in Figure 2.

#### Keywords

Together with lived experience advisors, researchers, and a Google Ads agent, we generated 4 lists of keywords: *low risk* keywords, which included keywords people are likely to search when in distress or situations associated with suicide, without explicitly mentioning suicide (eg, “Feeling so alone” and “debt”); *high risk* keywords, which included keywords explicitly communicating suicidal ideation or intent (eg, “I want to die”); *help seeking* keywords, which included keywords explicitly searching for help for suicidal thoughts (eg, “suicide help”); and *means specific* keywords, which relate to searching or using specific means.

#### Advertisements

**Figure 3:**
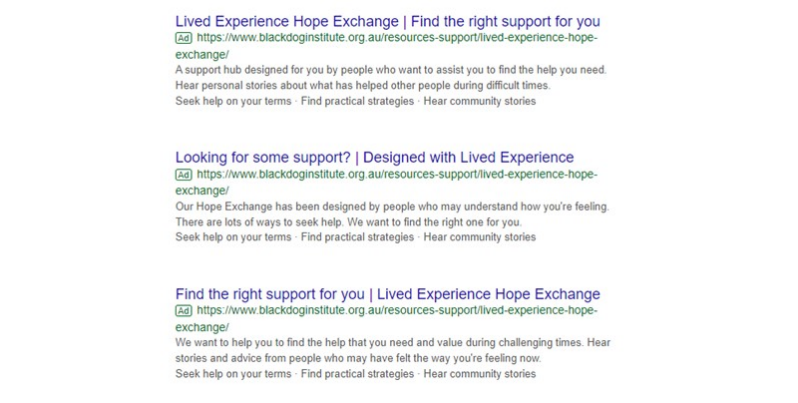
Advertisements shown on the Google search page if the advertisement is triggered in study 2.

Ads were shown to users independently of which category of keywords were searched. The co-design process yielded 3 similar advertisements. When the advertisement triggered, 1 of 3 advertisements would be randomly shown, resulting in equal presentations across the study. The 3 advertisements are shown in Figure 3.

#### Landing Page

In collaboration with lived experience advisors, we co-designed a series of landing pages with a primary focus on the contemplation stage, rather than crisis, which was entitled the “Lived Experience Hope Exchange.” The pages contained lived experience stories, calming and distracting activities, and links to support services and hotlines with descriptions of what the individual is likely to experience when engaging these services. In all, 4 different versions of the Hope Exchange were developed: a general version and 3 age-tailored versions (18-24, 25-44, and 45+ years). Details of the pages can be found elsewhere [10].

#### Randomization

Google Experiments, Google’s A/B testing feature, was used to randomize individuals clicking the ad link to either the general version of the Hope Exchange (50%) or the versions tailored to their estimated age (50%). We excluded any individual whose age could not be determined or who were aged <18 years. This randomization was implemented using Google Experiments; both participants and researchers were blinded.

#### Outcomes

The primary outcomes for study 2 were identical to study 1. However, given the much wider range of resources on the website compared to study 1, we included a wider range of conversions (drawing from engagement metrics in the advertising field) to include behaviors that the investigators, lived experience advisors, and collaborative team considered positive. Triggering any of these conditions was considered a conversion, including:

- Clicking the “get help” button to see available support services
- Downloading any file
- Clicking on a link to call a support service
- Spending more than 2 minutes on the website

#### Statistical Analysis

Data collection and analysis methods for study 2 were the same as for Study 1. Primary analyses included comparing the total conversion rates of studies 1 and 2 and the industry average; click rate by keywords searched; conversions by keywords searched; and conversions by tailoring condition.

#### Ethics Approval

This study was approved by the University of New South Wales Human Research Ethics Committee (HC210827), including a formal waiver of consent as it was not possible to obtain consent prior to participants searching for relevant keywords or clicking through on the displayed ads. The project did not meet the committee’s definition of a clinical trial, as it did not evaluate the effect of an intervention on health outcomes; as such, the study was not prospectively registered with the Australian New Zealand Clinical Trials Registry (ANZCTR).

### Results

The advertisements ran from March 2 to 21, 2022, in Australia with a total of 120,881 impressions, 6227 clicks (a 5.15% click-through rate), and 1419 conversions, with a conversion rate of 22.79% (1419/6227) and a total conversion rate of 1.17% (1419/120,881). The breakdown per keyword group is shown in Table 1. Note that due to the low number of the clicks related to the means-related keywords (n=4, with no conversions), these keywords were excluded from the analysis.

We investigated whether the study-2 total conversion rate was higher than those of study 1 or the industry standard. The results revealed that the study-2 campaign had a higher total conversion rate than the study-1 total conversion rate (IRD 0.005619, 95% CI 0.004984-0.007436; *P*<.001) and industry standard total conversion rate (IRD 0.01055, 95% CI 0.01036-0.01191; *P*<.001), with the study-2 total conversion rate being 1.91 times greater than the total conversion rate in study 1 and 9.75 times greater than the industry standard.

**Table 1:** Metrics from the study-2 Google Ads campaign.

Metric, campaign			Keyword type			
		Low risk		High risk	Help seeking	Means specific
**Impressions, n**						
	Total	108,219		3369	9079	223
	General	45,646		1800	5279	78
	Tailored (all)	62,573		1569	3800	145
	Tailored (18-24 years)	8837		311	683	26
	Tailored (25-44 years)	25,429		577	1274	72
	Tailored (45+ years)	28,307		681	1843	47
**Clicks, n**						
	Total	5530		118	575	4
	General	2440		56	340	2
	Tailored (all)	3093		62	235	2
	Tailored (18-24 years)	514		7	25	0
	Tailored (25-44 years)	1032		23	66	1
	Tailored (45+ years)	1547		32	144	1
**Click rate, n/N (%)**						
	Total	5530/108,219 (5.11)		118/3369 (3.5)	575/9079 (6.33)	4/223 (1.79)
	General	2440/45,646 (5.35)		56/1800 (3.11)	340/5279 (6.44)	2/78 (2.56)
	Tailored (all)	3093/62,573 (4.94)		62/1569 (3.95)	235/3800 (6.18)	2/145 (1.38)
	Tailored (18-24 years)	514/8837 (5.82)		7/311 (2.25)	25/683 (3.66)	0/26 (0)
	Tailored (25-44 years)	1032/25,429 (4.05)		23/577 (3.99)	66/1274 (5.18)	1/72 (1.39)
	Tailored (45+ years)	1547/28,307 (5.46)		32/681 (4.7)	144/1843 (7.81)	1/47 (2.13)
**Conversions, n**						
	Total	1209		37	171	0
	General	583		20	104	0
	Tailored (all)	626		17	67	0
	Tailored (18-24 years)	47		3	11	0
	Tailored (25-44 years)	185		7	19	0
	Tailored (45+ years)	394		7	37	0
**Conversion rate, n/N (%)**						
	Total	1209/5530 (21.86)		37/118 (31.36)	171/575 (29.74)	0/4 (0)
	General	583/2440 (23.9)		20/56 (35.71)	104/340 (28.51)	0/2 (0)
	Tailored (all)	626/3093 (20.25)		17/62 (27.42)	67/235 (30.59)	0/2 (0)
	Tailored (18-24 years)	47/514 (9.14)		3/7 (42.86)	11/25 (44)	0/0 (0)
	Tailored (25-44 years)	185/1032 (17.94)		7/23 (30.43)	19/66 (28.79)	0/1 (0)
	Tailored (45+ years)	394/1547 (25.49)		7/32 (21.88)	37/144 (25.69)	0/1 (0)
**Total conversion rate, n/N (%)**						
	Total	1209/108,219 (1.12)		37/3369 (1.1)	171/9079 (1.88)	0/223 (0)
	General	583/45,646 (1.28)		20/1800 (1.11)	104/5279 (1.97)	0/78 (0)
	Tailored (all)	626/62,573 (1)		17/1569 (1.08)	67/3800 (1.76)	0/145 (0)
	Tailored (18-24 years)	47/8837 (0.53)		3/311 (0.96)	11/683 (1.61)	0/26 (0)
	Tailored (25-44 years)	185/25,429 (0.73)		7/577 (1.21)	19/1274 (1.49)	0/72 (0)
	Tailored (45+ years)	394/28,307 (1.39)		7/681 (1.03)	37/1843 (2.01)	0/47 (0)

**Figure 4:**
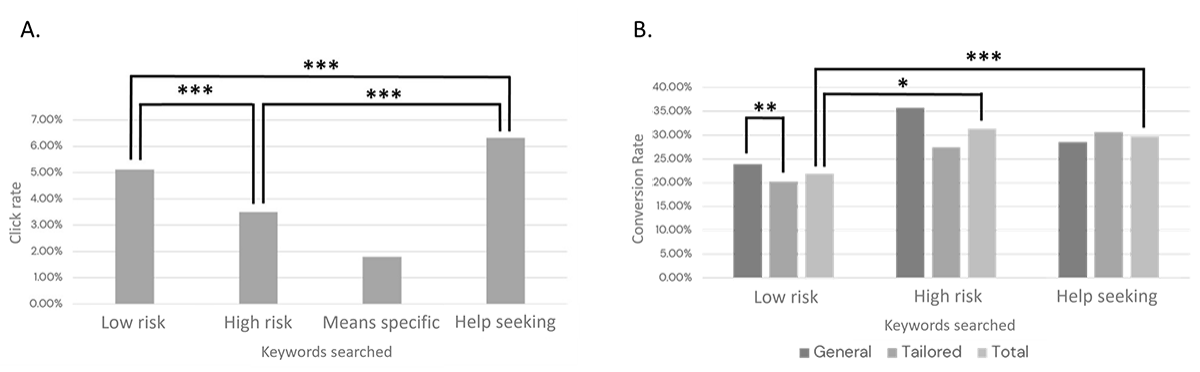
(A) Click through-rate by keywords searched. The x-axis shows the group of keywords searched. (B) Conversion rate by keywords searched. The x-axis shows the group of keywords searched, whereas different column shades indicate the trial tailoring condition. Total column is calculated using the following formula: (general click count + tailored click count) / (general conversion count + tailored conversion count). * Indicates *P*<.05, ** indicates *P*<.01, and *** indicates *P*<.001.

We then investigated whether the click-through rate differed as a function of keywords searched. The results revealed that the click rates for individuals searching *low risk* keywords, *high risk* keywords, and *help seeking* keywords were all significantly different from one another, in which individuals searching for *help seeking* keywords had the highest click rate, followed by individuals searching for *low risk* keywords, followed by individuals searching for *high risk* keywords. Results are shown in Figure 4A (low risk vs high risk: IRD 0.01608, 95% CI 0.00836-0.04194; *P*<.001; low risk vs help seeking: IRD 0.01223, 95% CI 0.001711-0.00734; *P*<.001; help seeking vs high risk: IRD 0.02831, 95% CI 0.01898-0.03764; *P*<.001).

We then investigated whether conversion rates differed by keyword type. The rate comparison analysis found a significant difference between *low risk* and *high risk* keywords (IRD 0.6972, 95% CI 0.00929-0.18058; *P*=.009), in which there was a higher conversion rate for people who searched for *high risk* keywords, and a significant difference between *low risk* and *help seeking* keywords (IRD 0.07877, 95% CI 0.03793-0.1196; *P*<.001), in which there was a higher conversion rate for individuals who are seeking help. However, there was not enough evidence to suggest that there was a difference in conversion rates between *high risk* and *help seeking* keywords (IRD 0.01617, 95% CI 0.09235-0.12469; *P*=.77). A graphical representation of these results can be found in Figure 4B.

Finally, we investigated whether age tailoring had a significant effect on conversions. The analysis revealed a significant difference between tailored and general landing pages for *low risk* keywords, in which general landing pages had a higher conversion rate than tailored landing pages (IRD 0.03635, 95% CI 0.06116-0.01153; *P*=.004). However, there was no significant difference for the general versus tailored landing pages for *high risk* (IRD 0.08295, 95% CI –0.11938 to 0.28528; *P*=.42) and *help seeking* keywords (IRD 0.02078, 95% CI 0.11145-0.0699; *P*=.65; see Figure 4B).

### Discussion

In this study, we codeveloped a series of search advertisement campaigns targeting individuals contemplating suicide and evaluated them for levels of reach and engagement. As per study 1, we observed high engagement relative to the industry standards. Within 21 days, we were able to reach individuals searching for suicide-related terms 120,881 times, with an average cost of US $13 each time a person engaged with a behavior designed to help them. Note that cost per conversion does not differ from one conversion to another; thus, it is the role of the Google Ads client—in this case, the research team—to set conversions that are meaningful.

Given that the advertisement was designed for individuals contemplating suicide, it is consistent with the finding that there was a significantly higher click-through rate for individuals searching for *low risk* than *high risk* keywords. The *help seeking* keywords yielded the highest click-through rate, which is consistent with the idea that individuals responded to the ad that featured words communicating explicitly that help could be found. This is consistent with the notion that the better the advertisement matches the search term, the more likely an individual is to click the advertisement. However, the analysis found a higher conversion rate for *high risk* compared to *low risk* keywords, which may stem from the fact that not all individuals searching for *low risk* keywords are searching for or need immediate help.

One finding was that there were markedly lower impressions, clicks, and conversions for individuals searching for *means specific* keywords, suggesting that overall, there were fewer people who were searching for *means specific* keywords. Given the low number of impressions and clicks, we were unlikely to see any conversions.

An unexpected finding was that there was a higher conversion rate for general landing pages compared to tailored landing pages for *low risk* keywords, suggesting that our tailoring was not effective in application and rather reduced engagement. This may indicate that despite there being clear, mutually exclusive preferences indicated by different age groups in the co-design process [10], these preferences may have limited generalizability or how we operationalized these preferences may be limited despite having approval from the co-design team. Other types of tailoring have yet to be explored.

## General Discussion

### Principal Findings

Across 2 studies, we investigated whether using Google Ads would allow us to reach individual searching for suicide-related terms for both crisis, in which we presented a suicide helpline, and contemplation, in which we presented a landing page offering help beyond presenting a helpline, such as hearing lived experience stories and calming exercises. Across both studies, we have observed exceptionally high click-through and conversion rates compared to the industry standard, with study-2 total conversion rate being 9.75 times higher than the industry standard. Overall, the data show that individuals are engaging well with both the advertisement and the pages themselves.

Given that for much of the time, it is likely that the hotline banners were present in both studies, future studies should investigate why individuals were still engaging with a search result if the helpline number was present on the search page. One possibility in study 1 is that within the few lines of text, the advertisement was able to provide more information on the hotline compared to the banner—that is, the hotline is anonymous and available 24/7. Lived experience advisors noted that it would be helpful to include descriptions on what it is like to call a hotline or engage in other services to help remove apprehension and promote help seeking [10]. In the second study, the *help seeking* keywords yielded the highest click-through rate—keywords that are likely to trigger a hotline banner given they explicitly indicate help seeking for suicide—further suggesting that there are individuals who are seeking help for suicide who do not want to immediately call a hotline, which is consistent with findings from the co-design study [10].

Future studies should investigate whether other advertising services can be used for suicide prevention. Although Google has a majority market share [12] for search engine advertisements, the advertising industry spans across multiple platforms. For example, there is data collection and advertising on social media (such as Twitter, Instagram, and TikTok) and streaming sites (eg, YouTube) and on advertising banners on websites (eg, Google AdSense). Future studies should investigate whether the findings from this study generalize to other platforms and whether using more than one platform for data collection and advertising increases reach and promotes help seeking. We propose, given the wealth of research showing web-based behaviors reflecting suicidality outside of search (eg, social media [19]), that we should see similarly fruitful findings outside of search engine advertisements.

Future work should also investigate how to integrate digital advertising such as Google Ads into routine practice. For example, given that individuals may discover existing resources through internet searches, partnerships with local health providers could help to place ads to link individuals to their local area’s most validated and well-resourced services. To implement this into routine practice, we would need to ascertain (1) the cost and cost-effectiveness of integrating digital advertising into existing health systems, (2) what services could be highlight on the landing pages, and (3) the funding requirements to sustain this approach.

### Limitations

Our investigation has several limitations. One limitation is that we did not record data on whether persons clicking on the ads were experiencing suicidal ideation or had engaged in suicidal behaviors, nor did we assess change in these outcomes. Future studies could investigate whether help-promoting web pages, such as those designed for this study, increase the number of individuals who seek help and reduce the suicide rate within a specific geographic region using Google Ads’ geographic targeting. Another limitation is that we do not know when the helpline was also triggered. Thus, we are unable to investigate how individuals behave when there is both the helpline and the advertisement, compared with just the advertisement.

Another limitation is that in study 2, we only included individuals whose ages could be ascertained by Google as 18 years or older. This was to ensure that the general versus age-tailored groups were comparable. However, we cannot determine what proportion of the population were excluded due to their ages being undetermined from past browsing history. Thus, future studies should also include individuals whose ages cannot be determined, where the analysis permits. Another key limitation in study 2 is that none of the advertisements explicitly used the word “suicide.” Although this is the result of the rigorous co-design process [10], there were advisors who suggested that in some settings, explicit use of the word “suicide” may lead to higher engagement. Thus, future studies should compare engagement with the campaign when advertisements use and do not use explicit suicide wording.

### Conclusion

Although the true effect of this intervention remains to be seen, the engagement metrics suggest that using internet ads may be useful in reaching out to individuals searching for suicide-related terms as a service delivery platform. Beyond suicidality, using internet ads may be helpful to reach out to individuals across a wide range of different health applications. Given that internet search is perhaps one of the most primary methods of information seeking and the ranking on search page can shape behavior, health-based organizations can use internet ads to ensure that what is presented is not only what is the most engaging but the most authoritative and valid.

## Appendix

Multimedia Appendix 1 CONSORT-eHEALTH checklist (V 1.6.2).

## Abbreviations

ANZCTR: Australian New Zealand Clinical Trials Registry
CONSORT: Consolidated Standards of Reporting Trials
IRD: incidence rate difference
