# Supplementary material for: Suicide Prevention Using Google Ads: Randomized Controlled Trial Measuring Engagement
Source: JMIR Ment Health. 2023 Apr 20;10:e42316. doi: 10.2196/42316 (PMC10160926; doi:10.2196/42316)
Supplement: Multimedia Appendix 1 [file mental_v10i1e42316_app1.pdf]

CONSORT-EHEALTH Checklist V1.6.2 Report	Manuscript Number	42316
**(based on CONSORT-EHEALTH V1.6), available at [http://tinyurl.com/consort-ehealth-v1-6].**		
**Date completed**		
1/24/2023 21:58:50		
**by**		
Sandersan Onie		
Suicide Prevention using Google AdWords: A Randomised Trial measuring Engagement		
TITLE		
**1a-i) Identify the mode of delivery in the title**		
The title contains "using Google AdWords" which in itself is an online advertising platform		
**1a-ii) Non-web-based components or important co-interventions in title**		
There are no non-web-based components in the intervention		
**1a-iii) Primary condition or target group in the title**		
The title has the words "Suicide Prevention" in the title		
ABSTRACT		
**1b-i) Key features/functionalities/components of the intervention and comparator in the METHODS section of the ABSTRACT**		
The features of the intervention can be found in the main body, but it is also present in the abstract: "First, we designed the campaign to focus on crisis, running a campaign for 16 days in which crisis related keywords would trigger an ad and landing page to help individuals find the national suicide hotline number. Second, we expanded the campaign to also help individuals contemplating suicide, running the campaign for 19 days with a wider range of keywords with a co-designed website with a wider range of offerings e.g., lived experience stories." The trial is the second study.		
**1b-ii) Level of human involvement in the METHODS section of the ABSTRACT**		
No, this item is irrelevant		
**1b-iii) Open vs. closed, web-based (self-assessment) vs. face-to-face assessments in the METHODS section of the ABSTRACT**		
No, this point is irrelevant		
**1b-iv) RESULTS section in abstract must contain use data**		
The abstract contains these wordings "In the second study, the ad was shown 120,881 times, clicked 6,227 times (5·15% click rate), with 1,419 engagements with the site (22·79%), a significantly higher rate than industry average of 3%. Clicks on ad was high despite a suicide hotline banner likely being present."		
**1b-v) CONCLUSIONS/DISCUSSION in abstract for negative trials**		
The primary outcome is discussed		
INTRODUCTION		
**2a-i) Problem and the type of system/solution**		
The problem is described here in "Previous studies have shown that individuals may search for suicide-related terms on the internet prior to an attempt. A recent study showed that in the 60-days prior to a suicide attempt, individuals had searched for terms expressing suicidal ideation, suicide means, but also relevant keywords not directly associated with crisis (e.g., feeling empty, divorce, and alcohol use) [1]. In addition, studies have found that the increases in the volume of internet searches for suicide-related terms predicted subsequent increases in national suicide rates [2,3,4]. Thus, targeting internet search engines to intervene in such searches, and connect individuals to help, may be an important avenue for intervention."		
**2a-ii) Scientific background, rationale: What is known about the (type of) system**		
The paper has the following regarding the system "One reason to use advertisements over organic (or non-advertisement) searches is that when using organic search, it is not guaranteed that a link will appear first on the search result page. A study found that on average, a link on the search page is twice more likely to be clicked on vs the link directly below it [6], suggesting that individuals are most likely to click and engage with the search result presented first. Further, organic search relies on similarities of the keyword and the page itself, thus a person who is searching for terms pertaining to "loneliness" may not be shown a link to a page with suicide help. Finally, internet searches are a part of daily life, with 93% of browsing sessions starting on the search page [7]; thus, individuals may be more likely to engage with help if the process by which they access help is a part of their day-to-day behaviour.<br>In some search engines, a hotline number will appear in a prominently displayed banner at the top of the search results if certain suicide-related terms are searched. Reports from the United States have shown that in certain instances, the banners have increased calls to the hotline by 10% [8]. However, there is no public information on what keywords will trigger the banner, and whether past search history increases or decreases the likelihood of this banner appearing. Further, both a Google spokesperson has said, and past research has shown that, not all relevant keywords will trigger this hotline banner [8,9] and not everyone in suicidal crisis would like to call a helpline [10]. Further, a phenomenon called 'banner blindness' is often observed, in which eye-tracking data suggests individuals will tend to ignore elements on a page in the form of a banner [11].<br>"		
**Does your paper address CONSORT subitem 2b?**		
The paper has noted the objectives here "In study 2, we developed a Google Ads campaign designed to be helpful for individuals who may be contemplating suicide, but not in immediate crisis [18]. In this context, we use the term contemplation phase to capture any stages which may lead to a suicide attempt, but prior to immediate crisis. Simply providing a link to the hotline may not be the most appropriate response in the contemplation stage, and therefore we undertook a co-design process to expand what was offered on the landing page. Further, given that the keywords searched may represent different cognitive states we investigated whether different categories of keywords led to different levels of engagement. Finally, we utilised Google AdWords' targeting features and investigated whether individuals show higher engagement with landing pages tailored to specific age groups compared to a general all-ages landing page. "		
METHODS		
**3a) CONSORT: Description of trial design (such as parallel, factorial) including allocation ratio**		
"The study employed a 2-arm experimental design (Landing Page: General vs. Age Tailored: 18 – 24, 25 – 44, 45+) with 4 initial pathways (individuals searching for different types of keywords: Low Risk, High Risk, Help Seeking, and Means Specific). Participants were allocated equally to the two arms."		
**3b) CONSORT: Important changes to methods after trial commencement (such as eligibility criteria), with reasons**		
N/A given there were no changes		
**3b-i) Bug fixes, Downtimes, Content Changes**		
N/A given there were no changes		
**4a) CONSORT: Eligibility criteria for participants**		
"Participants over the age of 18, whose ages could be inferred by Google from their past browsing history and are currently residing in Australia were eligible to join the trial. "		
**4a-i) Computer / Internet literacy**		
N/A, given that recruitment was done online, thus all participants are likely to have internet literacy		
**4a-ii) Open vs. closed, web-based vs. face-to-face assessments:**		
The study is completely online, over the internet.		
**4a-iii) Information giving during recruitment**		
"This study was approved by the University of New South Wales Human Research Ethics Committee, including a formal waiver of consent as it was not possible to obtain consent prior to participants searching for relevant keywords or clicking through on the displayed ads."		
**4b) CONSORT: Settings and locations where the data were collected**		
"Participants over the age of 18, whose ages could be inferred by Google from their past browsing history and are currently residing in Australia were eligible to join the trial."		
**4b-i) Report if outcomes were (self-)assessed through online questionnaires**		
We did not collect any information through questionnaires		
**4b-ii) Report how institutional affiliations are displayed**		
The landing page featured some, but minimal Black Dog Institute branding		
**5) CONSORT: Describe the interventions for each group with sufficient details to allow replication, including how and when they were actually administered**		
**5-i) Mention names, credential, affiliations of the developers, sponsors, and owners**		
The design process is reported in another upcoming paper "Onie S, Holland S, Elder E, Gale N, Finemore C, Livingstone N, Berlinquette P, Josifovski N, Shand F, Tye M & Larsen ME. Co-Design and Development of an Internet Search Intervention using Google AdWords for Individuals Contemplating Suicide: The Lived Experience Hope Exchange. In prep. "		
**5-ii) Describe the history/development process**		
The co-design process is briefly reported in Study 2's method section and in more detail in another paper "Onie S, Holland S, Elder E, Gale N, Finemore C, Livingstone N, Berlinquette P, Josifovski N, Shand F, Tye M & Larsen ME. Co-Design and Development of an Internet Search Intervention using Google AdWords for Individuals Contemplating Suicide: The Lived Experience Hope Exchange. In prep. "		
**5-iii) Revisions and updating**		
N/A there was not updating of the intervention beyond the initial version used at the start of the trial		
**5-iv) Quality assurance methods**		

N/A, we did not use quality assurance models		
**5-v) Ensure replicability by publishing the source code, and/or providing screenshots/screen-capture video, and/or providing flowcharts of the algorithms used**		
We do not provide the landing pages for this study as it is still being used for another investigation - people accessing it may compromise engagement metrics		
**5-vi) Digital preservation**		
We do not provide the landing pages for this study as it is still being used for another investigation - people accessing it may compromise engagement metrics		
**5-vii) Access**		
Participants accessed the landing page through the Google Search Page		
**5-viii) Mode of delivery, features/functionalities/components of the intervention and comparator, and the theoretical framework**		
This is reported in more detail in another upcoming paper ""Onie S, Holland S, Elder E, Gale N, Finemore C, Livingstone N, Berlinquette P, Josifovski N, Shand F, Tye M & Larsen ME. Co-Design and Development of an Internet Search Intervention using Google AdWords for Individuals Contemplating Suicide: The Lived Experience Hope Exchange. In prep. "		
**5-ix) Describe use parameters**		
Not relevant for the current intervention.		
**5-x) Clarify the level of human involvement**		
There was no human involvement in this trial.		
**5-xi) Report any prompts/reminders used**		
There was no prompts or reminders used in this intervention.		
**5-xii) Describe any co-interventions (incl. training/support)**		
There are no co-interventions		
**6a) CONSORT: Completely defined pre-specified primary and secondary outcome measures, including how and when they were assessed**		
"Data on impressions, clicks, click rate (clicks/impressions), conversions, conversion rate (conversion/click), cost per click, and cost per conversion was extracted from Google AdWords. Total conversion rate was manually calculated (conversions/impressions). The primary outcomes for this trial to measure engagement are click rate (engagement with the ad), conversion rate (engagement with the landing page), and total conversion rate (total engagement with campaign)."		
**6a-i) Online questionnaires: describe if they were validated for online use and apply CHERRIES items to describe how the questionnaires were designed/deployed**		
Outcomes were not due recorded through online questionnaires.		
**6a-ii) Describe whether and how "use" (including intensity of use/dosage) was defined/measured/monitored**		
Adoption was recorded with click, click-rate, conversion and conversion-rate.		
**6a-iii) Describe whether, how, and when qualitative feedback from participants was obtained**		
No qualitative feedback was recorded.		
**6b) CONSORT: Any changes to trial outcomes after the trial commenced, with reasons**		
"Participants over the age of 18, whose ages could be inferred by Google from their past browsing history and are currently residing in Australia were eligible to join the trial."		
**7a) CONSORT: How sample size was determined**		
**7a-i) Describe whether and how expected attrition was taken into account when calculating the sample size**		
No sample size calculation was present.		
**7b) CONSORT: When applicable, explanation of any interim analyses and stopping guidelines**		
"Data on impressions, clicks, click rate (clicks/impressions), conversions, conversion rate (conversion/click), cost per click, and cost per conversion was extracted from Google AdWords. Total conversion rate was manually calculated (conversions/impressions). The primary outcomes for this trial to measure engagement are click rate (engagement with the ad), conversion rate (engagement with the landing page), and total conversion rate (total engagement with campaign)."		
**8a) CONSORT: Method used to generate the random allocation sequence**		
"Google Experiments, Google's A/B testing feature was used to randomise individuals clicking the ad link to either the general version of the Hope Exchange (50%), or the version tailored to their estimated age (50%). We excluded any individual whose age could not be determined or who were under 18 years of age. This randomization was implemented using Google Experiments; both participants and researchers were blinded."		
**8b) CONSORT: Type of randomisation; details of any restriction (such as blocking and block size)**		
"Google Experiments, Google's A/B testing feature was used to randomise individuals clicking the ad link to either the general version of the Hope Exchange (50%), or the version tailored to their estimated age (50%). We excluded any individual whose age could not be determined or who were under 18 years of age. This randomization was implemented using Google Experiments; both participants and researchers were blinded."		
**9) CONSORT: Mechanism used to implement the random allocation sequence (such as sequentially numbered containers), describing any steps taken to conceal the sequence until interventions were assigned**		
"Google Experiments, Google's A/B testing feature was used to randomise individuals clicking the ad link to either the general version of the Hope Exchange (50%), or the version tailored to their estimated age (50%). We excluded any individual whose age could not be determined or who were under 18 years of age. This randomization was implemented using Google Experiments; both participants and researchers were blinded."		
**10) CONSORT: Who generated the random allocation sequence, who enrolled participants, and who assigned participants to interventions**		
"Google Experiments, Google's A/B testing feature was used to randomise individuals clicking the ad link to either the general version of the Hope Exchange (50%), or the version tailored to their estimated age (50%). We excluded any individual whose age could not be determined or who were under 18 years of age. This randomization was implemented using Google Experiments; both participants and researchers were blinded."		
**11a) CONSORT: Blinding - If done, who was blinded after assignment to interventions (for example, participants, care providers, those assessing outcomes) and how**		
**11a-i) Specify who was blinded, and who wasn't**		
All participants were blinded.		
**11a-ii) Discuss e.g., whether participants knew which intervention was the "intervention of interest" and which one was the "comparator"**		
No, participants were blinded and did not know.		
**11b) CONSORT: If relevant, description of the similarity of interventions**		
"In collaboration with lived experience advisors, we co-designed a series of landing pages with a primary focus on the contemplation stage, rather than crisis, which was entitled the 'Lived Experience Hope Exchange'. The pages contained lived experience stories, calming and distracting activities, and links to support services and hotlines with descriptions of what the individual is likely to experience when engaging these services. Four different versions of the Hope Exchange were developed: a general version, and three age-tailored versions (18-24, 25-44, and 45+). Details of the pages can be found elsewhere"		
**12a) CONSORT: Statistical methods used to compare groups for primary and secondary outcomes**		
"Data collection and analysis methods for Study 2 were the same as for Study 1. Primary analyses include comparing total conversion rate of Studies 1, 2, and the industry average; click rate by keywords searched, conversions by keywords searched, and conversions by tailoring condition."		
**12a-i) Imputation techniques to deal with attrition / missing values**		
Data does not have missing values		
**12b) CONSORT: Methods for additional analyses, such as subgroup analyses and adjusted analyses**		
"For any analyses in which we compare rates (e.g., click through rates or conversion rates), we used the MedCalc software which employs a chi-squared test to test a significant incidence rate difference (IRD) [16]. All rate comparisons in this paper use this method."		
RESULTS		
**13a) CONSORT: For each group, the numbers of participants who were randomly assigned, received intended treatment, and were analysed for the primary outcome**		
"The advertisements ran from 02/03/2022 to 21/03/2022 in Australia with a total of 120,881 impressions, 6,227 clicks, a 5·15% click through rate, 1,419 conversions with a conversion rate of 22·79% and total conversion rate of 1·17%. The breakdown per keyword group is shown in Table 1. Note due to the low number of the clicks related to the means related keywords (n=4, with no conversions), these keywords were excluded from the analysis. "		
**13b) CONSORT: For each group, losses and exclusions after randomisation, together with reasons**		
There is no consort diagram. "The advertisements ran from 02/03/2022 to 21/03/2022 in Australia with a total of 120,881 impressions, 6,227 clicks, a 5·15% click through rate, 1,419 conversions with a conversion rate of 22·79% and total conversion rate of 1·17%. The breakdown per keyword group is shown in Table 1. Note due to the low number of the clicks related to the means related keywords (n=4, with no conversions), these keywords were excluded from the analysis. "		
**13b-i) Attrition diagram**		
Individuals who went from impression, clicks to conversions are reported in Table 1.		
**14a) CONSORT: Dates defining the periods of recruitment and follow-up**		
"The advertisements ran from 02/03/2022 to 21/03/2022 in Australia with a total of 120,881 impressions, 6,227 clicks, a 5·15% click through rate, 1,419 conversions with a conversion rate of 22·79% and total conversion rate of 1·17%. The breakdown per keyword group is shown in Table 1. Note due to the low number of the clicks related to the means related keywords (n=4, with no conversions), these keywords were excluded from the analysis."		
**14a-i) Indicate if critical "secular events" fell into the study period**		
This was not recorded or reported.		
**14b) CONSORT: Why the trial ended or was stopped (early)**		

The trial ended as all funds were expended, as per prediction.		
**15) CONSORT: A table showing baseline demographic and clinical characteristics for each group**		
No demographic information is recorded		
**15-i) Report demographics associated with digital divide issues**		
No demographics are recorded; however, given that recruitment and intervention are done online, it can be assumed that all participants have access to internet.		
**16a) CONSORT: For each group, number of participants (denominator) included in each analysis and whether the analysis was by original assigned groups**		
**16-i) Report multiple “denominators” and provide definitions**		
All denominators are reported in the Study 2 results section.		
**16-ii) Primary analysis should be intent-to-treat**		
This analysis is not valid in this study.		
**17a) CONSORT: For each primary and secondary outcome, results for each group, and the estimated effect size and its precision (such as 95% confidence interval)**		
Confidence intervals are reported for all rate comparison analyses.		
**17a-i) Presentation of process outcomes such as metrics of use and intensity of use**		
This is not reported in the paper.		
**17b) CONSORT: For binary outcomes, presentation of both absolute and relative effect sizes is recommended**		
This is not reported in the paper.		
**18) CONSORT: Results of any other analyses performed, including subgroup analyses and adjusted analyses, distinguishing pre-specified from exploratory**		
"We investigated whether the Study 2 total conversion rate was higher than Study 1 or the industry standard. The results revealed that the Study 2 campaign had a higher total conversion rate than the Study 1 total conversion rate (IRD = 0·005619, p < ·0001, 95% CI [0·004984 – 0·007436]) and industry standard total conversion rate (IRD = 0·01055, p < ·0001, 95% CI [0·01036 – 0·01191]), with the Study 2 total conversion rate being 1·91 times greater than the total conversion rate in Study 1 and 9·75 times greater than the industry standard.<br>We then investigated whether click through rate differed as a function of keyword searched. The results revealed that the click rates for individuals searching low risk keywords, high risk keywords, and help seeking keywords all were significantly different from one another, in which individuals searching for help-seeking keywords had the highest click rate, followed by individuals searching for low-risk keywords, followed by individuals searching for high-risk keywords. Results are shown in Figure 4a (Low Risk vs. High Risk: IRD = 0·01608, p < ·0001, IRD 95% CI [0·00836 – 0·04194]; Low Risk vs. Help Seeking: IRD = 0·01223, p < ·0001, IRD 95% CI [0·001711 – 0·00734]; Help Seeking vs. High Risk: IRD = 0·02831, p < ·0001, IRD 95% CI [0·01898 - 0·03764]).<br>We then investigated whether conversion rates differed by keyword type. The rate comparison analysis found a significant difference between low risk and high-risk keywords (IRD = 0·6972, p = 0·009493, IRD 95% CI [0·00929 - 0·18058]) in which there was a higher conversion rate for people who searched for high-risk keywords, and a significant difference between low risk and help seeking keywords (IRD = 0·07877, p = 0·0002, IRD 95% CI [0·03793 - 0·1196]) in which there was a higher conversion rate for individuals who are seeking help. However, there was not enough evidence to suggest that there was a difference in conversion rates between high risk and help seeking (IRD = 0·01617, p = 0·7703, IRD 95% CI [0·09235 – 0·12469]). A graphical representation of these results can be found in Figure 4A.<br>Primary outcomes. Finally, we investigated whether age tailoring had a significant effect on conversions. The analysis revealed a significant difference between tailored and general landing pages for low-risk keywords in which general landing pages had a higher conversion rate than tailored landing pages (IRD = 0·03635, p = 0·0041, IRD 95% CI [0·06116 – 0·01153]). However, there was no significant difference for the general vs tailored landing pages for high risk (IRD = 0·08295, p = 0·4217, IRD 95% CI [-0·11938 – 0·28528]) and help seeking keywords (IRD = 0·02078, p = 0·6534, IRD 95% CI [0·11145 – 0·0699]), see Figure 4B.<br>"		
**18-i) Subgroup analysis of comparing only users**		
No subgroup analyses were conducted		
**19) CONSORT: All important harms or unintended effects in each group**		
No intended or unintended harms were recorded		
**19-i) Include privacy breaches, technical problems**		
No privacy or technical problems occurred during the trial		
**19-ii) Include qualitative feedback from participants or observations from staff/researchers**		
N/A, this information was not collected		
DISCUSSION		
**20) CONSORT: Trial limitations, addressing sources of potential bias, imprecision, multiplicity of analyses**		
**20-i) Typical limitations in ehealth trials**		
"Our investigation has several limitations. One limitation is that we did not record data on whether persons clicking on the ads were experiencing suicidal ideation or had engaged in suicidal behaviours, nor did we assess change in these outcomes. Future studies could investigate whether help-promoting webpages, such as those designed for this study, increases the number of individuals who seek help and reduces the suicide rate within a specific geographic region utilizing Google AdWords' geographic targeting. Another limitation is that we do not know when the helpline was also triggered. Thus, we are unable to investigate how individuals behave when there is both the helpline and the advertisement, compared with just the advertisement.<br>Another limitation is that in Study 2, we only included individuals whose ages could be ascertained by Google as 18 years or above. This was to ensure that the general vs. age tailored groups were comparable. However, we cannot determine what proportion of the population were excluded due to their ages being undetermined from past browsing history. Thus, future studies should also include individuals whose ages cannot be determined, where the analysis permits. Another key limitation in Study 2 is that none of the advertisements explicitly used the word 'suicide'. While this is the result of rigorous co-design process [10], there were advisors who suggested that in some settings, explicit use of the word 'suicide' may lead to higher engagement. Thus, future studies should compare engagement with the campaign when advertisements use and do not use explicit suicide wording. "		
**21) CONSORT: Generalisability (external validity, applicability) of the trial findings**		
**21-i) Generalizability to other populations**		
"Future work should also investigate how to integrate digital advertising such as Google AdWords into routine practice. For example, given that individuals may discover existing resources through internet searches, partnerships with local health providers could help to place ads to link individuals to their local area's most validated and well-resourced services. To implement this into routine practice, we would need to ascertain a) the cost and cost-effectiveness of integrating digital advertising into existing health systems, b) what services could be highlight on the landing pages, and c) the funding requirements to sustain this approach."		
**21-ii) Discuss if there were elements in the RCT that would be different in a routine application setting**		
We have noted that further work is needed to make this claim.		
**22) CONSORT: Interpretation consistent with results, balancing benefits and harms, and considering other relevant evidence**		
**22-i) Restate study questions and summarize the answers suggested by the data, starting with primary outcomes and process outcomes (use)**		
"In this study, we co-developed a series of search advertisement campaigns targeting individuals contemplating suicide and evaluated them for levels of reach and engagement. As per Study 1, we observed high engagement relative to the industry standards. Within 21 days, we were able to reach individuals searching for suicide-related terms 120,881 times, with an average cost of USD$13 each time a person engaged with a behaviour designed to help them. Note that cost per conversion does not differ from one conversion to another, thus it is the role of the Google AdWords client – in this case the research team – to set conversions that are meaningful.<br>Given that the advertisement was designed for individuals contemplating suicide, it is consistent with the finding that there was a significantly higher click through rate for individuals searching low-risk than high-risk keywords. The help-seeking keywords yielded the highest click through rate consistent with the idea that individuals responded to the ad which featured words communicating explicitly that help could be found. This is consistent with the notion that the better the advertisement matches the search term, the more likely an individual is to click the advertisement. However, the analysis found a higher conversion rate for high-risk compared to low-risk keywords, which may stem from the fact that not all individuals searching for low-risk keywords are searching for or need immediate help.<br>One finding was that there was markedly lower impressions, clicks and conversions for individuals searching for means-specific keywords, suggesting that overall, there were fewer people who were searching for means-specific keywords. Given the low number of impressions and clicks, we were unlikely to see any conversions.<br>An unexpected finding was that there was a higher conversion rate for general landing pages compared to tailored landing pages for low-risk keywords, suggesting that our tailoring was not effective in application and rather reduced engagement. This may indicate that despite there being clear, mutually exclusive preferences indicated by different age groups in the co-design process [10], these preferences may have limited generalizability, or how we operationalised these preferences may be limited despite having approval from the co-design team. Other types of tailoring have yet to be explored.<br>"		
**22-ii) Highlight unanswered new questions, suggest future research**		

"Future studies should investigate whether other advertising services can be used for suicide prevention. While Google has a majority market share [12] for search engine advertisements, the advertising industry spans across multiple platforms. For example, there is data collection and advertising on social media (such as Twitter, Instagram, and Tik Tok), streaming sites (e.g., YouTube) and on advertising banners on websites (e.g., Google AdSense). Future studies should investigate whether the findings from this study generalize to other platforms, and whether utilizing more than one platform for data collection and advertising increases reach and promoting help seeking. We propose, given the wealth of research showing online behaviours reflecting suicidality outside of search (e.g., social media [19]), that we should see similarly fruitful findings outside of search engine advertisements.<br>Future work should also investigate how to integrate digital advertising such as Google AdWords into routine practice. For example, given that individuals may discover existing resources through internet searches, partnerships with local health providers could help to place ads to link individuals to their local area's most validated and well-resourced services. To implement this into routine practice, we would need to ascertain a) the cost and cost-effectiveness of integrating digital advertising into existing health systems, b) what services could be highlight on the landing pages, and c) the funding requirements to sustain this approach.<br>"		
Other information		
**23) CONSORT: Registration number and name of trial registry**		
The trial was retrospectively registered at the Australia and New Zealand Clincal Trial Registry ACTRN12623000084684		
**24) CONSORT: Where the full trial protocol can be accessed, if available**		
There is no full trial protocol		
**25) CONSORT: Sources of funding and other support (such as supply of drugs), role of funders**		
"Study 1 was funded by Patrick Berlinquette. Funding for study 2 was obtained by Sandersan Onie, Mark Larsen, Michelle Tye and Fiona Shand."		
**X26-i) Comment on ethics committee approval**		
"This study was approved by the University of New South Wales Human Research Ethics Committee, including a formal waiver of consent as it was not possible to obtain consent prior to participants searching for relevant keywords or clicking through on the displayed ads."<br><br>"Google AdWords has several ethical protections in place. The data is only available in aggregate format, and it is not possible to identify individual people who have seen the ad, clicked on the ad, or browse the landing page website. While Google AdWords may use past browsing history to present advertisements that the individual is likely to engage with, the user can erase all data Google has collected [13] or opt out of personalized advertising [14]. Furthermore, personal information, such as email, is never collected or shared without express permission [15]. Ethics approval was not required for this study as only de-identified, pre-existing data are reported in aggregate."		
**x26-ii) Outline informed consent procedures**		
"This study was approved by the University of New South Wales Human Research Ethics Committee, including a formal waiver of consent as it was not possible to obtain consent prior to participants searching for relevant keywords or clicking through on the displayed ads."		
**X26-iii) Safety and security procedures**		
"Google AdWords has several ethical protections in place. The data is only available in aggregate format, and it is not possible to identify individual people who have seen the ad, clicked on the ad, or browse the landing page website. While Google AdWords may use past browsing history to present advertisements that the individual is likely to engage with, the user can erase all data Google has collected [13] or opt out of personalized advertising [14]. Furthermore, personal information, such as email, is never collected or shared without express permission [15]. Ethics approval was not required for this study as only de-identified, pre-existing data are reported in aggregate."		
**X27-i) State the relation of the study team towards the system being evaluated**		
The evaluator and authors also designed the intervention		
